# Sex and ethnic differences in the cardiovascular complications of type 2 diabetes

**DOI:** 10.1177/20420188211034297

**Published:** 2021-08-04

**Authors:** Jian L. Yeo, Emer M. Brady, Gerry P. McCann, Gaurav S. Gulsin

**Affiliations:** Department of Cardiovascular Sciences, University of Leicester and the Leicester NIHR Biomedical Research Centre, Glenfield Hospital, Groby Road, Leicester, LE3 9QP, UK; Department of Cardiovascular Sciences, University of Leicester and the Leicester NIHR Biomedical Research Centre, Glenfield Hospital, Leicester, UK; Department of Cardiovascular Sciences, University of Leicester and the Leicester NIHR Biomedical Research Centre, Glenfield Hospital, Leicester, UK; Department of Cardiovascular Sciences, University of Leicester and the Leicester NIHR Biomedical Research Centre, Glenfield Hospital, Leicester, UK

**Keywords:** sex differences, ethnic differences, type 2 diabetes, cardiovascular complications

## Abstract

Diabetes mellitus represents a global health concern affecting 463 million adults and is projected to rapidly rise to 700 million people by 2045. Amongst those with type 2 diabetes (T2D), there are recognised differences in the impact of the disease on different sex and ethnic groups. The relative risk of cardiovascular complications between individuals with and without T2D is higher in females than males. People of South Asian heritage are two to four times more likely to develop T2D than white people, but conversely not more likely to experience cardiovascular complications. Differences in the pathophysiological responses in these groups may identify potential areas for intervention beyond glycaemic control. In this review, we highlight key differences of diabetes-associated cardiovascular complications by sex and ethnic background, with a particular emphasis on South Asians. Evidence assessing therapeutic efficacy of new glucose lowering drugs in minority groups is limited and many major cardiovascular outcomes trials do not report ethnic specific data. Conversely, lifestyle intervention and bariatric surgery appear to have similar benefits regardless of sex and ethnic groups. We encourage future studies with better representation of women and ethnic minorities that will provide valuable data to allow better risk stratification and tailored prevention and management strategies to improve cardiovascular outcomes in T2D.

## Introduction

Diabetes mellitus represents a major global health concern with rapidly increasing prevalence. In 2019, an estimated 9.3% (463 million people) of the world’s population aged 20–79 years had diabetes. This is projected to rise to 10.9% (700 million people) by the year 2045.^1^ The vast majority (90%) have type 2 diabetes (T2D), most commonly associated with obesity and characterised by a combination of insulin resistance, hyper- and hypo-insulinaemia and subsequent hyperglycaemia. Despite the multi-organ involvement of T2D, cardiovascular diseases (CVD) account for the most deleterious consequences of T2D. In a systematic review of over 4.5 million people worldwide with T2D, CVD affected almost one-third and accounted for half of all deaths.^2^

Amongst people with T2D, there are recognised differences in its CVD impact on different sex and ethnic groups. For example, the global estimated prevalence of diabetes is comparable between women and men (9.0% *versus* 9.6%),^1^ yet the relative risk of cardiovascular (CV) complications between individuals with and without T2D is higher in females than males. Similarly, people of South Asian heritage are two to four times more likely to develop T2D than white people.^3^ However, the pathophysiology of diabetes complications in sex and ethnic groups is incompletely understood. In this review, we highlight key differences of diabetes CV complications related to sex and ethnicity, with a particular emphasis on the South Asians in the United Kingdom (UK). We summarise the pathophysiological mechanisms of these differences and evidence from major clinical studies to date to explore the heterogeneous effect of T2D amongst these groups. Lastly, we highlight studies identifying sex and ethnic differences in therapeutic response to novel glucose lowering agents. While our focus is on T2D, we found that many studies of T2D population did not report sex- or ethnicity-specific outcome data. We have, therefore, included some references with study populations other than T2D but with conditions closely associated with T2D (e.g. hypertensive or obese populations) that provided sex- or ethnicity-specific data.

## Sex differences in CV complications

**Figure 1.**
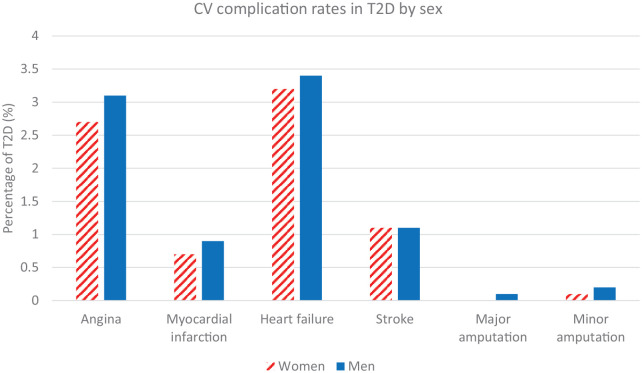
CV complication rates in 1.47 million women and 1.82 million men with T2D. Outcomes data from hospital admissions in England and Wales, National Diabetes Audit 2017–2018. CV, cardiovascular; T2D, type 2 diabetes.

Although the overall incidence of CVD in the general population and in people with diabetes is higher in men (Figure 1),^4,5^ large scale population studies comparing people with and without T2D overwhelmingly show that the relative risks for major CVD are up to 57% higher in women compared with men, even after controlling for common confounding variables such as age, obesity, smoking status, and blood pressure (BP) (Table 1). The greater relative risk in women is seen across all age groups but is higher among those below 60 years.^5^ In the absence of diabetes, it appears that women benefit from protection against CVD – a survival advantage that is lost with the development of diabetes, when their CVD burden approaches levels seen in men. Various hypotheses have been proposed to explain the ‘disadvantage’ accrued by women with diabetes, although reasons for this remain to be fully elucidated.^6^

**Table 1.** Population studies of sex differences in CV complications in T2D.

First author	Study design, country	Study details	Outcome measure	Relative risk of CVD in T2D versus euglycemia (95% CI)		
				Female	Male	Female:male ratio^a^
Emerging Risk Factors Collaboration.^7^	Meta-analysis of 102 prospective studies, multiple countries	*n* = 698,782, 43% F T2D *n* = 39,295 (6%) Age: 52 ± 13 years Follow up: median 10.8 years Adjustment: age, smoking, BMI, SBP	Coronary heart disease (fatal or non-fatal)	2.59 (2.29–2.93)	1.89 (1.73–2.06)	1.37 (*P*_interaction_ <0.0001)
			Ischaemic stroke	2.83 (2.35–3.40)	2.16 (1.84–2.52)	1.31 (*P*_interaction_ 0.0089)
Peters *et al.*^8^	Meta-analysis of 64 prospective studies, multiple countries	*n* = 858,507, 53% F T2D *n* = 35 359 (4%) Age: >18 years Follow up: 5–30 years Adjustment: multiple adjusted	Coronary heart disease (fatal or non-fatal)	2.63 (2.27–3.06)	1.85 (1.64–2.10)	1.44 (1.27–1.63)
Peters *et al.*^9^	Meta-analysis of 64 prospective studies, multiple countries	*n* = 775,385, 53% F T2D *n* = 33 772 (4%) Age: >15 years Follow-up: 5–32 years Adjustment: multiple adjusted	Stroke	2.28 (1.93–2.69)	1.83 (1.60–2.08)	1.27 (1.10–1.46)
Shah *et al.*^10^	CALIBER prospective study, England	*n* = 1,921,260, 51% F T2D *n* = 34,198 (2%) Age: >30 years Follow up: median 5.5 years Adjustment: age, BMI, socioeconomic status, HDL and total cholesterol, SBP, smoking, statin and antihypertensive drug	Non-fatal MI^b^	2.68 (2.03–3.54)	1.71 (1.45–2.45)	1.57 (*P*_interaction_ 0.0048)
			PAD^b^	5.11 (4.05–6.44)	3.77 (3.21–4.44)	1.36 (*P*_interaction_ 0.028)
Millett *et al.*^4^	UK Biobank prospective study, UK	*n* = 471,998, 56% F T2D *n* = 20 483 (4%) Age: 56 ± 8 years Follow up: mean 7 years Adjustment: age, SBP, socioeconomic status, smoking, BMI, lipid lowering and antihypertensive drug	Coronary heart disease (fatal or non-fatal)	1.96 (1.60–2.41)	1.33 (1.18–1.51)	1.47 (1.16–1.87)
Ohkuma *et al.*^11^	Meta-analysis of 47 prospective studies, multiple countries	*n* = 11,925,128 T2D *n* = 676 016 (6%) Age: >16 years Follow up: 3–27 years Adjustment: multiple adjusted	Congestive heart failure	1.95 (1.70–2.22)	1.74 (1.55–1.95)	1.09 (1.05–1.13)
Wright *et al.*^12^	Retrospective CPRD registry study between 2006–2013, England	Cases Female: *n* = 35,396, age 64 ± 14 Male: *n* = 44,589, age 61 ± 13 Controls Female: *n* = 172,994, age 64 ± 14 Male: *n* = 213,553, age 61 ± 13 Follow up: mean 3.6 years Adjustment: age, ethnicity, deprivation, smoking, obesity, co-morbidities	MACE (composite of MI, ischaemic stroke, CV death)	1.20 (1.12–1.28)	1.12 (1.06–1.19)	1.07 (0.98–1.17)
			MI	1.31 (1.20–1.43)	1.20 (1.12–1.28)	1.09 (0.98–1.22)
			Ischaemic stroke	1.13 (1.01–1.26)	1.04 (0.92–1.16)	1.09 (0.93–1.28)
Malmborg *et al.*^5^	Retrospective registry study between 2012–2016, Denmark	Cases Female: *n* = 69,057, age 65 years (56–74) Male: *n* = 79,328, age 64 years (55–71) Controls Female: *n* = 1,266,291, age 57 years (48–67) Male: *n* = 1,132,245, age 55 years (47–65)	MACE-HF (composite of MI, ischaemic stroke, HF, CV death)	2.90 (2.90–2.90)	2.50 (2.40–2.50)	1.15 (1.11–1.19)
			MI	2.70 (2.60–2.90)	2.00 (2.00–2.10)	1.34 (1.25–1.43)
			Ischaemic stroke	2.40 (2.30–2.50)	2.20 (2.10–2.20)	1.09 (1.03–1.15)
			HF	3.60 (3.40–3.70)	3.20 (3.10–3.30)	1.13 (1.07–1.19)
			All-cause mortality	2.70 (2.70–2.80)	2.60 (2.60–2.70)	1.03 (1.00–1.06)
Chase-Vilchez *et al.*^13^	Meta-analysis of seven prospective studies, multiple countries	*n* = 2,071,260, 50% F T2D *n* = 39,569 (2%) Age: 45–72 years Follow-up: 5–20 years Adjustment: multiple adjusted	PAD	1.96 (1.29–2.63)	1.84 (1.29–2.86)	1.05 (0.90–1.22)

Age presented as median (interquartile range) or mean ± SD.

a Where provided, *p* values for interaction with sex are presented. In meta-analyses, pooled estimates of women:men relative risk ratio were generated using random effects analysis with inverse variance weighting.

b In participants younger than 60 years.

BMI, body mass index; CALIBER, CArdiovascular disease research using LInked Bespoke studies and Electronic health Records; CI, confidence interval; CPRD, Clinical Practice Research Datalink; CV, cardiovascular; CVD, cardiovascular disease; DBP, diastolic blood pressure; HDL, high-density lipoprotein; HF, heart failure; MACE, major adverse cardiovascular events; MI, myocardial infarction; PAD, peripheral arterial disease; SBP, systolic blood pressure; SD, standard deviation; T2D, type 2 diabetes; UK, United Kingdom.

### Atherosclerotic CV disease

#### Coronary artery disease

Coronary artery disease is the leading cause of death globally, with a higher overall prevalence in men than women in general population.^14^ However, when comparing between people with and without diabetes, the relative risk of coronary disease is 40–50% higher in women (Table 1). For example, a retrospective analysis of hospital admissions for CVD in England (*n* = 2,572,566, 37% female) comparing individuals with and without diabetes, reported the presence of diabetes in women conferred a greater excess risk of hospital admission for myocardial infarction (MI) compared with men [incidence rate ratio (IRR) of women versus men: 4.27 versus 3.15]. Furthermore, women with diabetes were more likely to require percutaneous coronary intervention (IRR 4.37 versus 3.14) or coronary artery bypass graft surgery (IRR 6.24 versus 5.01).^15^ The INTERHEART study showed that the first presentation of MI is approximately 9 years later in women than men without diabetes.^16^ The younger age of first MI in men is explained largely by a more adverse risk factor profile, including abnormal lipids and smoking. T2D, however, brings forward the onset of MI and stroke by 20–30 years in women compared with 15–20 years in men.^17^ Furthermore, outcomes following MI are worse in women than men with T2D. In a Swedish cohort of post-MI patients (*n* = 25,555, 18% T2D, 23% female, mean age 56 years) followed for up to 8 years showed that women had higher mortality than men [relative risk (RR) 1.34, 95% confidence interval (CI) 1.16–1.55], which was attributed to greater burden of co-morbidities such as hypertension.^18^

Importantly, as well as having a disproportionate excess risk of coronary artery disease associated with diabetes, the clinical manifestation of coronary artery disease is different between women and men. Women more frequently present with atypical symptoms of angina,^19^ which may hamper accurate diagnosis and early treatment, therefore potentially leading to disease progression and consequent MI.

Interestingly, diabetes is inversely associated with development of thoracic and abdominal aortic aneurysms.^20^ This paradoxical protective effect of diabetes against aneurysmal formation is thought to be mediated by atherosclerotic process within the aortic vessel wall, including decreased mural neo-angiogenesis, intraluminal thrombus formation, inflammation, extracellular matrix remodelling, and vascular smooth muscle cell alterations.^20^ The net effect results in smaller aortic diameter and lower risk of rupture. Although highly topical, the sex and ethnic differences of aortic atherosclerotic disease in T2D is beyond the scope of this review.

#### Cerebrovascular disease

The excess risk of stroke conferred by diabetes is greater in women than men, a pattern consistent with diabetes-associated coronary disease. This is evidenced by two large meta-analyses, which have shown that when compared with people without T2D, women with T2D have approximately one-third higher risk of stroke events than men (Table 1).^7,9^ The excess risk persists despite adjusting for other major CV risk factors.

#### Peripheral arterial disease

Studies examining sex differences in the prevalence of peripheral artery disease (PAD) in diabetes differ between studies, with some studies supporting a stronger association of diabetes with PAD in women, while others have shown that the risk is similar in both sexes. For example, the Framingham study (*n* = 5209, 55% female) reported that, in people with impaired glucose tolerance, the risk of intermittent claudication is doubled in men, but quadrupled in women.^21^ In the presence of glycosuria, this risk is magnified to 8.6-fold in women compared with 3.5-fold in men.^21^ However, a recent meta-analysis of PAD risk in diabetes showed no overall difference between women and men (RR 1.96 *versus* 1.84),^13^ in contrast to the greater excess risk seen in women for other diabetes-related CVD (Table 1).

Importantly, women have been under-represented in clinical trials of PAD, comprising only about a third of study populations.^22^ Similar to coronary artery disease, women present with PAD at an older age. Women are more likely than men to be asymptomatic or have atypical symptoms of PAD, leading to delayed presentation, when they are more likely to have critical limb ischaemia.^23^

Women with T2D and PAD benefit less from exercise rehabilitation for treatment of claudication compared with men, showing lesser improvements in claudication onset time and walking distance.^24^ T2D is also associated with greater post-surgical mortality in women than in men. Following infra-inguinal bypass for PAD, 3-year survival was significantly lower in women (54% *versus* 72%, *p* < 0.05).^25^ Furthermore, women with T2D had 2.5 times higher mortality rate compared with women without T2D, while there was no significant difference between men with and without T2D.

### Heart failure

Although atherosclerotic diseases are typically regarded as the predominant manifestation of CVD in people with T2D, heart failure is increasingly being recognised as a major cause of morbidity and mortality. Diabetes is an independent risk factor for heart failure, even after accounting for the presence of other risk factors.^26^ T2D is associated with both common forms of heart failure: heart failure with reduced ejection fraction (HFrEF) or preserved ejection fraction (HFpEF), although HFpEF appears to be the predominant manifestation of heart failure in diabetes. Heart failure affects around 15% of people with T2D, and it appears that T2D is a stronger risk factor for heart failure in women than men.^2^ The largest meta-analysis of population-based cohorts to date included more than 12 million individuals (6% had T2D) and showed the excess risk of heart failure in T2D is 9% higher in women than men (Table 1).^11^

**Figure 2.**
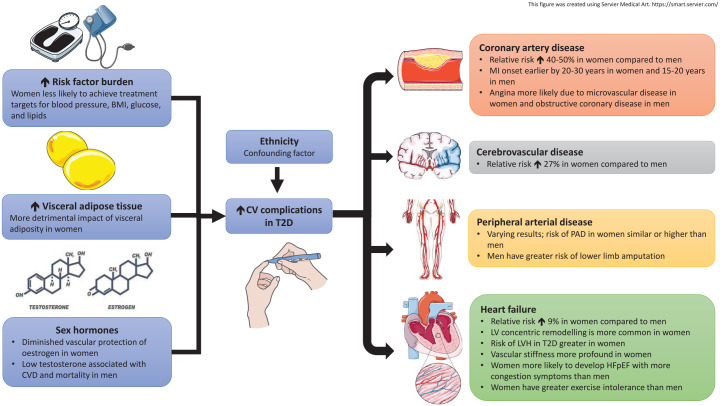
Sex differences in CV complications in T2D. The greater relative risks of T2D-associated CV complications among women than men are attributed to worse cardiometabolic profile, greater impact of visceral adiposity and diminished vascular protection of oestrogen in women. Coronary microvascular disease and HFpEF are also more prevalent among women with T2D. BMI, body mass index; CVD, cardiovascular disease; HFpEF, heart failure with preserved ejection fraction; MI, myocardial infarction; PAD, peripheral arterial disease; T2D, type 2 diabetes.

The pathophysiology of diabetic cardiomyopathy is complex and has been studied extensively in humans *in vivo* using advanced non-invasive cardiac imaging techniques. Typically, early subclinical cardiac alterations [e.g. left ventricular (LV) remodelling, diastolic dysfunction, diffuse myocardial fibrosis and microvascular dysfunction] are detected and have been associated with increased risk of progression to overt symptomatic heart failure.^27^ Moreover, sex differences in energy metabolism (lower glucose uptake and greater fatty acid utilisation in females), nitric oxide signalling, calcium handling (lower calcium transient and contractile reserve in females),^28^ and influence of sex hormones on hyperglycaemic pathways have been implicated the pathophysiology of diabetic cardiomyopathy.^29^ These could all account for differences in the phenotypic expression of heart failure in men and women with diabetes and further studies are needed to identify key mechanisms driving differential heart failure risk in men and women with T2D. The sex differences in CV complications are summarised in Figure 2.

## Sex differences in potential mechanisms of CV complications in T2D

### Clinical and metabolic risk factors

A possible explanation for the excess CV risk in women with T2D may be the differential impact of concomitant clinical and metabolic risk factors commonly associated with diabetes. Hypertension, smoking and diabetes were associated with higher hazard ratios for MI in women,^4^ and women with coronary events were more likely to have three or more CVD risk factors than men.^30^ However, in a Finnish study (*n* = 2131, 53% female, 39% T2D), despite adjusting for smoking, body mass index (BMI), systolic BP and lipids, the excess risk for diabetes-related coronary heart disease events remained higher in women [hazard ratio (HR) 9.5 in women and 2.8 in men], suggesting that T2D drives CVD independently of traditional risk factors.^31^

Multiple studies have reported disparities in risk factor control in T2D between sexes. Data from the British Regional Heart Study and British Women’s Heart and Health (*n* = 7529, 50% females, mean age 69 years) found that although women had more favourable risk factor profile than men in normoglycaemia, this deteriorated to a greater degree relative to men in T2D. Specifically, women with T2D had worse clinical (waist circumference, BMI, diastolic BP) and biochemical markers of inflammation (white blood cell count), lipids, insulin resistance and coagulation (von Willebrand factor, factor VIII) compared with men.^32^ Furthermore, consistent reports of poorer modifiable risk factor profile in women with T2D, especially greater obesity and lower achievement of glycaemia and lipid targets, have been seen in multiple countries including Italy,^33^ Netherlands,^34^ and the United States (US).^35^ Interestingly, all these studies reported that utilisation of lipid-lowering, glucose-lowering and anti-hypertensive medication among women were either greater than or comparable with men. Proposed mechanisms of a lower achievement of treatment targets in women include worse cardiometabolic risk factor profile in women at initiation of pharmacotherapy, lower prescribed doses, poorer adherence to medication, and sex-difference in the physiological drug response.^33^ They suggested that women with diabetes should receive more aggressive glucometabolic risk factor reduction than men, which may help lower the excess burden of CVD in women with T2D.

### Adipose tissue distribution

Body fat distribution varies physiologically between sexes. Women, in response to oestrogen, favour fat storage in the subcutaneous tissues and lower extremities. Conversely, men tend to accumulate a greater proportion of visceral fat.^36^ Increased visceral fat is associated with insulin resistance, hyperglycaemia and increased hepatic free fatty-acid delivery.^37^ Consequently, women need to gain more weight than men to attain a proportional increase of visceral fat before the state of diabetes is achieved.^38^

Visceral adipose tissue (VAT) secretes cytokines, which are known to mediate inflammation, myocardial fibrosis and microvascular dysfunction, processes pivotal in the pathophysiology of CVD.^39^ Moreover, VAT has differential effect on cardiometabolic risk and appears to have greater detrimental impact in women than men. For example, in a case-control study of 105 individuals with HFpEF, increased VAT was associated positively with increased pulmonary capillary wedge pressure, a haemodynamic marker of HFpEF, in women but not in men.^40^ In the Framingham Heart Study (*n* = 1155, mean age 63 years, 55% women), pericardial adiposity was associated more strongly with dysglycaemia and metabolic syndrome in women despite men having a greater amount of pericardial fat.^41^

### Cardiovascular effects of sex hormones

#### Oestrogen

There is established evidence that oestrogen has CV protective properties.^42^ These include direct effects on the endothelium, regulation of gene expression to enhance production of collagen and angiogenic growth factors and reducing lipid concentrations.^42^ Diabetes seems to overcome these protective mechanisms of oestrogen and may partly explain the excess risk of CVD in pre-menopausal women.

In an analysis of patients undergoing coronary angiography from the Women’s Ischaemia Syndrome Evaluation cohort (*n* = 95), hypothalamic hypoestrogenaemia was more common among premenopausal women with than without T2D (50% *versus* 26%, *p* = 0.02).^43^ Moreover, the presence of both hypoestrogenaemia and T2D was associated with increased prevalence and severity of coronary artery disease compared with either of these conditions alone.^43^ While limited by selection bias, this study provided evidence of a possible link between hyperglycaemia, premature oestrogen deficiency, loss of oestrogen-mediated protection and subsequent increased risk of atherosclerosis.

Menopausal transition is recognised to accelerate CVD risk in women.^44^ While women have an overall greater excess CVD risk associated with T2D than men, the steepest progression of this excess risk occurs from the age of 40 years and peaking between 50 and 60 years,^5^ coinciding with the onset of menopause and diminishing oestrogen levels compounding the CV impact of T2D.

Oestrogen has been implicated in the regulation of vascular health but treating its deficiency is clearly more complex. Laboratory experiments have demonstrated beneficial responses at the cellular level and early observational studies of hormonal replacement seem to support a reduction in the incidence of CVD. However, randomised controlled trials (RCTs) failed to show a clear benefit in post-menopausal women. Of note, the trials were not designed to evaluate hard CV endpoints, but instead used surrogate markers, with conflicting results.^45^ It is also important to consider the harmful effects associated with exogenous hormone replacement including risk of breast cancer, arrhythmias and venous thrombosis.^46^ Oestrogen replacement, therefore, may be indicated for treatment of peri-menopausal symptoms (within 5 years of menopause) but not recommended solely for prevention of CVD based on current evidence.^47^

#### Testosterone

Testosterone promotes lipolysis, lean muscle mass and better glycaemic control in men. There are also additional benefits towards quality of life and sexual function.^48^ Hypogonadism is present in a third of men with diabetes, leading to testosterone deficiency.^48^ A meta-analysis (*n* = 16,709) has suggested that a higher testosterone level reduces the risk of developing T2D in men (RR 0.65, 95% CI 0.50–0.84, *p* = 0.001).^49^ Conversely, the effect of testosterone differs in women. While associated with lower risk of T2D in men, a higher testosterone level increased the risk of T2D (OR 1.37, 95% CI 1.22–1.53) and polycystic ovary syndrome in women, as shown in a UK Biobank study that genetically determined testosterone levels in over 425,000 participants.^50^

Low testosterone levels are associated with increased CVD and mortality. In a retrospective study of 857 men with T2D, testosterone replacement therapy was associated with lower all-cause mortality compared with those who were untreated (HR 0.38, 95% CI 0.16–0.90).^51^ However, heterogeneity in study designs and inconsistent results to date have not provided clarification to the effect of testosterone replacement therapy in CV risk reduction in men with diabetes.^52^

### Arterial stiffness and hypertension

Arterial stiffening increases with age in both men and women but a more rapid rise is seen in post-menopausal women,^53^ suggesting the interaction between oestrogen and vascular compliance. This is consistent with data that shows prevalence of hypertension in women begins to overtake men following onset of menopause.^54^ Diabetes and metabolic syndrome have been shown to be associated with aortic stiffening. These conditions have a more profound effect on vascular stiffness in women than men, showing greater increases in pulse wave velocity – a measure of arterial stiffness.^55^ Furthermore, aortic stiffening has been shown to be correlated with concentric LV remodelling and diastolic dysfunction on magnetic resonance imaging (MRI), suggesting a potential link with heart failure development.^56^

### Microvascular dysfunction

In those diagnosed with angina, women had less angiographically significant coronary artery disease, with microvascular dysfunction suggested as being the possible cause of angina.^57^ This suggests that, although macro and microvascular coronary disease affect both sexes, men more commonly present with epicardial coronary plaque events while women have a tendency towards vasospasm and microvascular dysfunction linked to impaired endothelial function. Importantly, abnormal coronary flow reserve, even in absence of significant epicardial disease, is associated with increased risk of major adverse CV outcomes in women.^58^

In a retrospective analysis of T2D individuals without obstructive epicardial coronary disease who underwent invasive vasoreactivity testing (*n* = 129, 60% female, mean age 50 years), Sara *et al.* reported that abnormal microvascular function was associated with poor glycaemic control (HbA1c > 7%) in women (OR 1.69, 95% CI 1.01–2.86), but not in men.^59^ Despite large trials previously showing that intensive glucose control did not reduce major CV outcomes,^60,61^ the evidence from this study suggests that stricter glycaemic control still has a role in preventing microvascular complications, at least in women, and underscores the need to tailor risk management according to sex. The authors also speculated that the under-representation of women in these major diabetes trials (proportion of male participants ranging 58% to 97%) may explain the negative result in part due to sex selection bias.^59^

### Cardiac remodelling

Numerous studies have demonstrated that diabetes is associated with alterations in LV geometry, typically increased LV mass and reduced chamber volumes, leading to concentric LV remodelling.^62^ The association of diabetes and LV hypertrophy appears stronger in women than men. In a cohort of hypertensive patients (*n* = 550, 44% female, 36% T2D, mean age 66 years) who underwent echocardiography, the age-adjusted risk for increased LV mass index in T2D versus non-T2D was significantly higher in women (RR 1.47, 95% CI 1.0–2.2) compared with men (RR 0.8, 95% CI 0.5–1.3).^63^ Furthermore, progression of LV hypertrophy differs between women and men; women preferentially develop a concentric rather than eccentric pattern of hypertrophy in response to hypertension and obesity – both risk factors that frequently accompany diabetes.^64^ Indeed, compared with an eccentric morphology, concentric remodelling or hypertrophy heralds a more unfavourable prognosis.^65^ This predisposition to concentric LV hypertrophy may explain the higher proportion of women with T2D who develop HFpEF, a condition also associated with concentric LV remodelling.

### Systolic and diastolic dysfunction

In a meta-analysis of echocardiographic studies comprising over 6000 people with T2D, the overall prevalence of (symptomatic or asymptomatic) LV diastolic dysfunction was 46% (95% CI 39–54%), and was similar in women and men (47% and 46%, respectively).^66^ Out of 28 studies included, only one (*n* = 605) compared sex differences in the prevalence of symptomatic HFpEF, which was more common among women than men (28% versus 18%, respectively). By contrast, a second meta-analysis from the same group (17 studies, *n* = 7542) found the overall prevalence of (symptomatic or asymptomatic) LV systolic dysfunction in T2D was 13% (95% CI 13–14%).^67^ Sex-specific data (only available for seven studies) showed systolic dysfunction was less common in women compared with men (0.1% *versus* 7%). Only one study compared prevalence of symptomatic HFrEF, which was higher among men.^67^ From these data it appears that, although the prevalence of diastolic dysfunction is similar in men and women with T2D, women have a higher predisposition towards developing symptoms of HFpEF. This may be because women with diabetes exhibit higher LV diastolic resistance and filling pressure than men, as demonstrated by more prominent increase in echocardiographic LV filling pressures (E/e’) during exercise echocardiography.^68^ Sex differences in cardiac adaptions to exercise may explain the higher prevalence of HFpEF in women with T2D, although further longitudinal studies are required.

### Myocardial fibrosis

Diffuse myocardial fibrosis in T2D has been reported widely both in animal models and human studies,^69^ and is thought to be mediated by damage from oxidative stress, inflammation, advanced glycation end-products and apoptosis.^62^ Gadolinium-enhanced cardiac MRI allows calculation of myocardial extra-cellular volume (ECV) – a marker of diffuse interstitial fibrosis. Importantly, a higher ECV has been shown to be associated with impaired myocardial function, heart failure and mortality in T2D.^70^

Sex difference of myocardial fibrosis has been demonstrated in non-diabetes CV diseases, although results are conflicting, with some showing more or less fibrosis on MRI.^71,72^ However, imaging studies addressing the sex difference of myocardial fibrosis specific to T2D are lacking. Interestingly, one animal study has shown diabetic female rats had significant cardiomyocyte hypertrophy without fibrosis, while male rats developed fibrosis but not hypertrophy.^73^ This disparity suggests a heterogenous mechanism at play in the development of myocardial fibrosis that is sex- and disease-dependent, warranting further evaluation in dedicated T2D cohorts.

## Ethnic differences in complications of T2D

This review focusses on South Asians, referred to as individuals who are immigrants or descendants from the Indian subcontinent including Bangladesh, Bhutan, India, Maldives, Nepal, Pakistan and Sri Lanka. According to the England and Wales census in 2011, South Asians constituted approximately 5% of the UK population, with Asian Indian and Pakistani origin being the largest groups.^74^ Multiple epidemiological studies have shown that the prevalence of T2D is 2–4 fold higher in South Asians than in white ethnic groups.^75,76^ This is corroborated by figures from the International Diabetes Federation, which reported that the age-adjusted prevalence of T2D in 2019 in South Asians is up to five times higher than the UK (Table 2). South Asians also have an earlier age of onset of T2D by a decade than white people.^77^

**Table 2.** Prevalence of T2D in South Asian countries and in UK. Source: International Diabetes Federation.^78^

Country	Number of adults 20–79 years with T2D	National age-adjusted prevalence in adults 20–79 years (%)
Bangladesh	8,372,000	9.2
India	77,006,000	10.4
Maldives	23,000	9.2
Nepal	697,000	7.2
Pakistan	19,370,000	19.9
Sri Lanka	1,233,000	10.7
UK	2,681,000	3.9

T2D, type 2 diabetes; UK, United Kingdom.

### Mechanisms of increased risk of T2D in South Asians

The mechanisms explaining why South Asians are at higher risk of developing diabetes have been reviewed extensively in articles by Shah and Kanaya and Sattar and Gill.^77,79^ South Asians develop diabetes at a lower BMI than white Europeans.^80^ Accordingly, lower cut-off values of 23 kg/m^2^ and 27.5 kg/m^2^ have been recommended to identify South Asians at increased or high risk of T2D, respectively.^81^ Biological factors contributing to increased T2D risk among South Asians include lower lean body mass, higher percentage of abdominal subcutaneous and visceral adiposity,^82^ greater insulin resistance, adipocyte dysfunction and more rapid decline in pancreatic beta-cell function with aging.^83^ Lifestyle factors include high-calorie diet and lower physical activity,^84,85^ both associated with urbanisation and migration into affluent western societies. Other risk factors reported were lower socioeconomic status in adults and children, less education and chronic psychological burden.^86^

### Prognosis of CVD in South Asians with T2D

**Figure 3.**
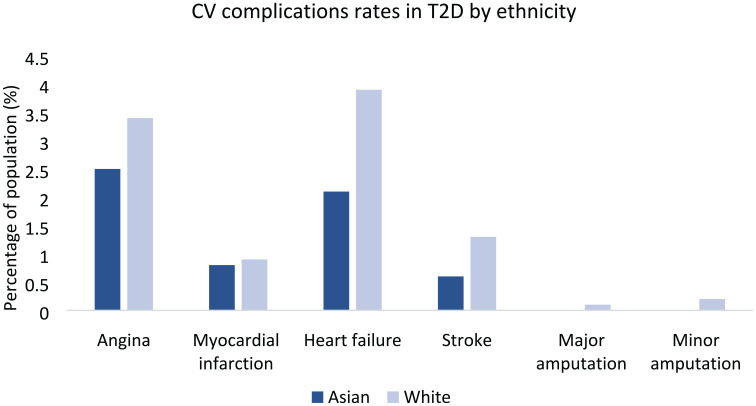
CV complication rates in T2D by ethnicity. Outcomes data from hospital admissions in England and Wales, National Diabetes Audit 2017–2018, show that white British have higher rates of T2D-associated CV complications than Asians. CV, cardiovascular; T2D, type 2 diabetes.

Despite an increased risk of T2D, associated mortality among South Asians has declined substantially since the 1990s with lower rates compared with white Europeans. This may be due to improved diabetes prevention through earlier screening and treatment, better risk factor management and healthcare awareness among second generation migrants.^87^ Additionally, amongst T2D, South Asians do not appear to be at higher risk of CV morbidity than white individuals, with some studies reporting lower relative risk for stroke and PAD in South Asians (Table 3). Indeed, the latest UK National Diabetes Audit 2017–2018 (*n* = 3,293,965) revealed that white British had highest rates of hospitalisation for angina, heart failure and stroke compared with other ethnicities (Figure 3).^88^

**Table 3.** Studies of CV complications in South Asians and whites with T2D.

First author	Study design, country	South Asian		White		Follow up, years	Adjustments	Outcome	Incidence rate per 1000 person-years		Adjusted RR/HR/OR of South Asian versus white (95% CI)
		*n*	Age, years^a^	*n*	Age, years^a^				South Asian	White	
Bellary *et al.*^89^	UKADS prospective study, UK	1486	57 ± 12	492	65 ± 12	2	Age, sex, diabetes duration, BP, lipids, HbA1c, smoking, and medication	Composite of coronary heart disease, stroke, or peripheral arterial disease	26.1	19.3	1.40^b^ (0.90–2.20)
Khan *et al.*^90^	Retrospective registry study between 1993–2007, Canada	15,066	57 ± 12	244,017	61 ± 13	Median 4	Age, socioeconomic status, province, and 16 other comorbidities	MI^c^	7.9	7.9	1.05 (0.92–1.19)
								Stroke^c^	4.3	5.1	**0.82 (0.68–0.99)**
								Heart failure^c^	4.4	6.4	**0.71 (0.56–0.92)**
								All-cause mortality^c^	20.5	32.6	**0.68 (0.63–0.74)**
Shah *et al.*^91^	Retrospective registry study between 2002–2009, Canada	22,342	52 ± 14	448,255	58 ± 15	Median 4.7	Age, sex, socioeconomic status, hypertension, comorbidities, and primary care visits before diabetes diagnosis	Coronary artery disease	13.8	13.7	1.01 (0.95–1.07)
								Stroke	3.2	3.9	**0.82 (0.72–0.94)**
								Lower extremity amputation	0.2	0.7	**0.31 (0.19–0.49)**
								Any CV complication	16.8	17.9	0.95 (0.90–1.00)
								All-cause mortality	12.6	22.2	**0.56 (0.52–0.60)**
Davis *et al.*^92^	UKPDS 83 prospective study, UK	418	47 ± 8	3543	53 ± 9	Median 18	Age, sex, BMI, smoking status, social class, waist circumference, lipids, HbA1c and systolic blood pressure	MI	13.2	16.6	1.11 (0.96–1.28)
								Stroke	3.3	6.0	0.98 (0.78–1.23)
								Peripheral arterial disease	0.3	2.3	**0.43 (0.23–0.82)**
								Diabetes-related death	7.2	14.8	0.90 (0.79–1.03)
								Any diabetes-related end point	43.2	45.2	1.18 (1.07–1.29)
								All-cause mortality	12.4	27.1	**0.89 (0.80–0.97)**
Sebastianski *et al.*^93^	Meta-analysis of seven studies, UK	4296	55 ± 6	18,539	61 ± 5	Not reported	Age and sex (but not in all included studies)	Peripheral arterial disease	0.5	1.8	**0.44 (0.30–0.63)**
Wright *et al.*^94^	Retrospective CPRD registry study between 1998–2015, England	9523	53 ± 14	143,724	63 ± 14	Mean 5	Age, sex, ethnicity, deprivation, and calendar year	CV mortality	6.1	16.4	**0.82 (0.75–0.89)**
								All-cause mortality	14.7	45.8	**0.70 (0.65–0.76)**

a Age presented as mean ± SD.

b Fatal and non-fatal CVD events.

c Values presented for men only.

BP, blood pressure; CI, confidence interval; CPRD, Clinical Practice Research Datalink; CV, cardiovascular; CVD, cardiovascular disease; HR, hazard ratio; MI, myocardial infarction; OR, odds ratio; RR, relative risk; SD, standard deviation; THIN, The Health Improvement Network; UKPDS, United Kingdom Prospective Diabetes Study; UK, United Kingdom; UKADS, United Kingdom Asian Diabetes Study.

### Atherosclerotic CVD in South Asians with T2D

#### Coronary artery disease

In a large survey by the British Cardiovascular Intervention Society (*n* = 280,000, 7% South Asians), it was found that South Asians were more likely to have multi-vessel coronary disease, corresponding to the higher prevalence of diabetes affecting 42% of South Asians compared with 15% of white Caucasians.^95^ South Asians also presented with coronary disease approximately 5 years earlier, likely in parallel to younger age of diabetes onset. In a case–control comparison of MI between participants from South Asia (*n* = 3936, 44% cases) and other countries (*n* = 23,159, 46% cases) in the global INTERHEART study, South Asians had higher rates of MI at younger ages (53 years versus 59 years, *p* < 0.001), attributed to greater risk factor levels, including dyslipidaemia and diabetes.^96^

Comparing cohorts from the MESA and MASALA studies, which comprised asymptomatic individuals free of CVD, a similar coronary calcium burden was seen in South Asians and white Americans in general.^97^ However, diabetes seemed to have a greater effect on progression of coronary calcification in South Asians. This was corroborated by a Dutch study of South Asians with T2D (*n* = 120, 45% women, mean age 52 years) compared with matched Caucasians that reported that South Asians were more likely to have coronary calcification, higher calcium scores and obstructive coronary artery disease, particularly involving the left anterior descending artery.^98^ However, data from larger cohort studies do not seem to suggest a difference in the incidence of coronary events between South Asians and white Europeans with T2D (Table 3). The disconnect between higher prevalence and extent of coronary disease but no increase of MI events warrants further evaluation but may reflect that calcified lesions are more stable than non-calcified plaques.^99^

#### Cerebrovascular disease

While there is an increased risk of stroke with T2D, this excess risk does not appear to be greater in South Asians when compared with white Europeans – a result consistent with findings for coronary disease. A comparable stroke risk in migrant South Asians was found between South Asians and Europeans with T2D in the UK Prospective Diabetes Study (UKPDS 83) long-term follow up, and population studies from Canada reported around 20% lower risk in South Asians with diabetes compared with whites (Table 3).^90–92^

#### Peripheral arterial disease

South Asians have lower prevalence of PAD than white Europeans, in both general and diabetes populations. The reasons for this are unclear and in spite of equivalent burden of coronary disease, which is not explained by other risk factors including obesity, lipids and glycaemic status.^100^ Commensurately, South Asians also have about threefold lower risk of diabetic foot ulcers or lower limb amputation.^91,101^ Reasons for this disparity despite higher prevalence of diabetes among South Asians have yet to be elucidated. Some proposed explanations include lower prevalence of smoking and higher premature death rate associated with T2D among South Asians before developing significant PAD.^102^ In contrast, a recent article by Armengol *et al*. (Dutch *n* = 165, South Asian *n* = 591) reported no difference in rates of PAD between the native Dutch population and South Asian Surinamese minority (6.7% *versus* 10.8%, *p* = 0.156; OR 0.91, 95% CI 0.39–2.08).^103^ Overall, South Asians are not disadvantaged with regards to the excess risk of PAD associated with T2D compared with white people.

### Heart failure

**Figure 4.**
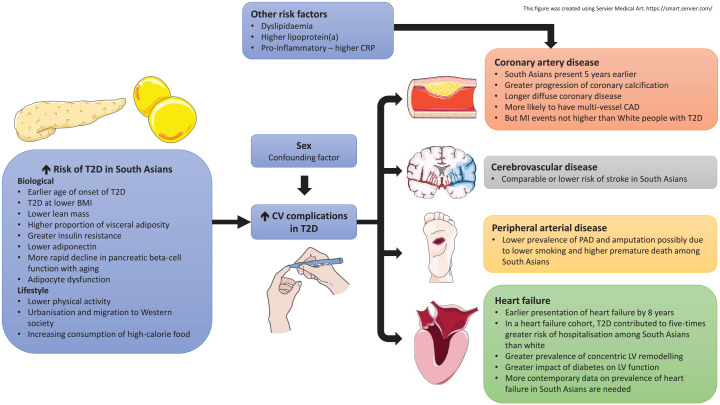
Differences in CV complications in South Asians compared with white Europeans with T2D. A combination of biological and lifestyle factors is recognised to increase the risk of T2D among South Asians, predisposing them to CV morbidities. South Asians with T2D present with more severe coronary disease and heart failure at an earlier age than white Europeans. However, epidemiological studies of people with T2D show that prevalence of MI and stroke are comparable between white people and South Asians, while prevalence of peripheral arterial disease may be lower in South Asians. BMI, body mass index; CAD, coronary artery disease; CRP, C-reactive protein; CV, cardiovascular; LV, left ventricular; MI, myocardial infarction; PAD, peripheral arterial disease; T2D, type 2 diabetes.

As the first generation migrant South Asians approach older age, the rates of heart failure presentation are expected to rise. In a historical cohort study between 1998 to 2001 of newly diagnosed heart failure (including people with and without diabetes) conducted in Leicestershire, UK (*n* = 5789, 6% South Asians), South Asians had up to 5-fold greater hospital admission rates for heart failure compared with whites (rate ratio 5.2 for women and 3.8 for men).^104^ Additionally, South Asians were on average 8 years younger and had greater prevalence hypertension (44% versus 29%), diabetes (46% versus 16%) or MI (27% versus 15%) than whites. However, despite more co-morbidity, risk of all-cause death at up to 3 years follow up was lower in South Asians (HR 0.82, 95% CI 0.68–0.99). Several reasons for this were proposed, including less severe heart failure at the point of admission, differing causes of heart failure or better family support post-hospitalisation among South Asians. Nevertheless, more recent data on the prevalence and outcomes of heart failure specifically among South Asians is limited. One study reported the risk of heart failure in diabetes was lower in South Asians than in white Europeans, albeit statistically significant only for men (Table 3).^90^ Further contemporary studies are warranted to confirm if heart failure outcomes among South Asians with T2D have improved over time. The differences in CV complications between South Asians and white Europeans are summarised in Figure 4.

### Ethnic differences in the potential mechanisms of CV complications in T2D

#### Dyslipidaemia and inflammation

In addition to T2D, greater dyslipidaemia is seen in South Asians. For example, analysis of 160,000 individuals comparing lipid profiles of ethnic minorities with white Americans showed South Asians were twofold more likely to have high triglycerides (OR 2.10 for women and 2.62 for men, *p* < 0.001) and threefold more likely to have low HDL (OR 3.90 for women and 2.94 for men, *p* < 0.001), while odds for high LDL were slightly increased (OR 1.16 for women, and 1.31 for men, *p* < 0.001).^105^ Increased atherogenicity and pro-inflammatory state, characterised by higher serum lipoprotein(a) and C-reactive protein, have also been reported in South Asians.^106,107^

#### Cardiac remodelling in South Asians

Differences in the cardiac structure and function between South Asians and white Europeans have been identified in the general population (Table 4). South Asians have demonstrated increased prevalence of LV hypertrophy, with a threefold increase in Indian compared with European men, in keeping with the higher prevalence of diabetes and hypertension among Asian Indian population, although this was not seen in women.^108^ The increased risk of LV hypertrophy persists after adjusting for clinical, metabolic and haemodynamic variables, indicating there is another yet unknown reason for the LV hypertrophy beyond diabetes. Additionally, concentric LV remodelling pattern was more evident in both South Asian men and women compared with white Europeans (adjusted relative wall thickness for men: 0.41 versus 0.39, *p* < 0.001; women: 0.40 versus 0.38, *p* < 0.01).^108^ Diabetes is also associated independently and more strongly with impaired LV function in South Asians than Europeans.^109^ This may be related to a greater burden of hypertension and diabetes in South Asians, and could explain their higher risk of heart failure development.

**Table 4.** Imaging studies of cardiac structure and function in South Asians compared with Europeans.

First author	Study groups		Key inclusion/exclusion	Modality	Main findings
	South Asians	Europeans			
Chahal *et al.*^110^	*n* = 259, 47% F Age: 51* *±* *9* *years	*n *=* *199, 40% F Age: 52* *±* *9* *years	Healthy subjects with no hypertension, dyslipidaemia, T2D, smoking or coronary disease. LOLIPOP cohort, UK	2D echocardiography	↓ left heart volumes and ↓ LV mass index in Indian Asians.
					↑ concentric remodelling in Indian Asians (relative wall thickness 0.37 *versus* 0.35, *p* < 0.001) independent of age, sex, BP, and BMI.
					↓ LV function in Indian Asians, demonstrated by ↓ s’ (8.9 ± 1.5 cm/s *versus* 9.5 ± 1.6 cm/s), ↓ e’, (10.3 ± 2.1 cm/s *versus* 11.0 ± 2.1 cm/s) and ↑ E/e’ ratio (7.9 ± 2.1 *versus* 7.0 ± 1.5), all *p* < 0.001.
Chahal *et al.*^108^	*n* = 1159, 33% F Age: 57 ± 10 years, 5–9% T2D	*n *=* *968, 26% F Age: 57 ± 10 years, 16–21% T2D	General population with no clinical cardiovascular disease. LOLIPOP cohort, UK	2D echocardiography	↑ prevalence of LV hypertrophy in Indian Asian men (adjusted OR 2.8, 95% CI 1.9–4.2) but not in women (adjusted OR 1.1, 95% CI 0.6–2.2).
					↑ concentric remodelling in Indian Asians (adjusted RWT for men 0.41 *versus* 0.39, *p* < 0.001; women 0.40 *versus* 0.38, *p* < 0.01).
					↓ longitudinal systolic function and diastolic function in Indian Asians (lower s’ and e’ and higher E/e’, all *p* < 0.05 Indian Asians *versus* Europeans).
Park *et al.*^111^	*n* = 325, 12% F Age: 69 ± 7 years, 38% T2D	*n* = 427, 22% F Age: 70 ± 6 years,15% T2D	First generation migrant South Asian. SABRE cohort, UK	3D echocardiography	↓ LV mass in South Asians when indexed to height^2.7^ (28.1 ± 0.4 g/m^2.7^ *versus* 29.8 ± 0.3 g/m^2.7^) or body surface area (61.9 ± 0.7 g/m^2^ *versus* 66.1 ± 0.6 g/m^2^), all *p* < 0.01. Findings persisted after adjusting for cardiometabolic risk factors.
					↓ LV remodelling index (LV mass/volume) in South Asians (1.47 ± 0.02 *versus* 1.52 ± 0.02, *p* < 0.05) independent of cardiometabolic risk factors.
Park *et al.*^109^	*n* = 457, 15% F Age: 69 ± 6 years, 41% T2D	*n* = 542, 23% F Age: 70 ±6 years, 18% T2D	First generation migrant South Asians. SABRE cohort, UK	2D echocardiography	↑ adverse effect of T2D on South Asians than Europeans with worse diastolic (E/e’ beta 0.69 ± 0.12 *versus* 0.09 ± 0.2, *p* for HbA1c/ethnicity interaction = 0.005) and systolic function (s’ beta −0.11 ± 0.06 *versus* 0.14 ± 0.09, *p* interaction = 0.2).
					Multivariable adjustment for hypertension, microvascular disease, LV mass, coronary disease and dyslipidaemia only partially accounted for the ethnic differences.
Paiman *et al.*^112^	T2D: *n* = 33, 64% F Age: 51 ± 9 years Controls: *n* = 21, 71% F Age: 48 ± 8 years	T2D: *n* = 48, 42% F Age: 60 ± 7 years Controls: *n* = 29, 48% F Age: 58 ±8 years	Overweight T2D, no history of significant coronary or valvular disease, no NYHA III-IV heart failure	Cardiac MRI at three Tesla and proton-magnetic resonance spectroscopy	↑ LV concentric remodelling and ↓ diastolic function (lower E/A ratio) and seen in both South Asians and Europeans with T2D.
					In T2D versus controls South Asians: ↑ LV mass (93 ± 20 *versus* 66 ± 15 g), myocardial cell volume (66±16 *versus* 45 ± 11 ml), and extracellular volume (23 ± 5 *versus* 18 ± 4 ml) but ↓ extracellular volume fraction (26.2 ± 3.0 *versus* 28.2 ± 2.6%). All *p* < 0.05.
					In T2D versus controls Europeans: ↑ myocardial triglyceride (1.19 ± 0.53 *versus* 0.58 ± 0.18%) and impaired LV global longitudinal strain (−19.3 ± 2.7 *versus* −21.1 ± 3.3%). All *p* < 0.05.

A, transmitral peak late diastolic flow velocity; E, transmitral peak early diastolic flow velocity; e’, mitral annular early diastolic velocity; LOLIPOP, London Life Science Prospective Population; LV, left ventricle; MRI, magnetic resonance imaging; NYHA, New York Heart Association; SABRE, Southall and Brent Revisited study; s’, mitral annular systolic velocity; T2D, type 2 diabetes.

A recent study by Paiman *et al*. revealed interesting results that further unravels phenotypic differences of diabetic hearts between South Asians (*n* = 54) and white Europeans (*n* = 77).^112^ Using cardiac MRI and proton magnetic resonance spectroscopy, they showed that, comparing people with and without T2D, white Europeans had higher myocardial triglyceride and reduced LV global longitudinal strain while South Asians had greater increases in LV mass, cellular volume and extracellular volume. These findings suggest that pathophysiological mechanisms in the development of diabetic cardiomyopathy and subsequent heart failure are potentially ethnicity-dependent and targeted therapies may be warranted.

## Sex and ethnic differences in therapy for T2D and CV outcomes

### Newer glucose-lowering therapies and CV outcomes

Compelling evidence from recent CV outcomes trials strongly support the benefits of novel glucose-lowering therapies for improving outcomes in people with T2D. The glucagon-like peptide-1 receptor agonist (GLP-1RA) and sodium-glucose co-transporter-2 inhibitors (SGLT-2i) have been shown to reduce adverse CV and renal adverse outcomes.^113,114^ Accordingly, the American Diabetes Association and European Association for the Study of Diabetes recommends GLP-1RA (if atherosclerotic disease predominates) or SGLT-2i (if heart failure or chronic kidney disease predominates) as second-line therapy in addition to metformin for management of hyperglycaemia, in those with established CVD. The mechanisms by which they improve CV outcomes, both in people with or without T2D, are still a subject of intense research.

#### SGLT-2 inhibitors

SGLT-2i reduce renal glucose reabsorption, thus enhancing glycosuria and lowering plasma glucose. Other notable effects include BP reduction and weight loss. Overall, there has been no demonstrated sex difference in the efficacy or safety of SGLT-2i. In a pooled analysis including four major trials comparing SLGT-2i with placebo in T2D (EMPA-REG OUTCOME, CANVAS, DECLARE TIMI-58, and CREDENCE; 34% female), there were no sex differences in reduction of major adverse CV events, hospitalisation for heart failure, CV death or total mortality (all *p* > 0.12 for sex interaction).^115^ Furthermore, both women and men had similarly increased risk of known side-effects, such as amputation or genital mycotic infection.^115^ More recently, Raparelli *et al*. compared newer glucose-lowering agents with sulphonylurea in over 167,000 T2D individuals (46% female, mean age 59 years) followed up for a median of 4.5 years.^116^ SGLT-2i again did not demonstrate a sex difference in CV outcomes or safety profile.

The limited data on ethnic differences in efficacy of SGLT-2i are conflicting. A meta-analysis of over 4000 participants evaluating SGLT-2i compared with placebo, has suggested greater glucose-lowering efficacy of SGLT-2i in Asian cohorts (HbA1c reduction by 0.96% versus 0.64%, *p* = 0.0003),^117^ while another suggested no difference.^118^ However, these meta-analyses included studies that were conducted in predominantly East Asian countries and therefore their results cannot be generalised to the South Asian population. There is no data on the effect of SGLT-2i on CV outcomes specific to South Asians, which is likely to reflect the relatively low numbers included in the trials.

#### GLP-1 receptor agonist

GLP-1 receptor agonist (GLP-1RA) is delivered by subcutaneous injection and acts by stimulating insulin and suppressing glucagon secretion. Additional effects include promoting weight loss by increasing satiety and reducing gastric emptying. Only a few studies of GLP-1RA have reported sex-specific CV outcomes. GLP-1RAs were better in reducing composite adverse CV outcomes than sulphonylurea, but this effect appears greater in women than men (HR 0.57 versus 0.82, *p* = 0.002).^116^ A meta-analysis of seven major trials involving GLP-1RA (*n* = 56,004, 37% female), however, reported equal reduction in major adverse CV events by 12% in both sexes when compared with placebo.^119^

There is some evidence that GLP-1RAs provides more substantial CV benefit in Asians compared with the white population. Specifically, in a subgroup analysis of three CV outcomes trials involving GLP-1RA (*n* = 27,389, 10% Asian), the rate of three-point major adverse CV events (CV death, MI, stroke) was reduced by about eightfold more in Asians than in white people (RR 0.35 versus 0.92, *p* < 0.001).^120^ The mechanisms for this observed difference are unclear but could reflect a greater effect on reducing inflammation in Asians that may lead to less plaque rupture.^121^

### Lifestyle interventions

There are clear benefits of exercise on CVD prevention and mortality in T2D in women and men.^122,123^ The LOOK-AHEAD trial (*n* = 5145, mean age 58.7 years, BMI 36 kg/m^2^, 60% females) compared the effects of intensive lifestyle intervention comprising of low-calorie diet and increased physical activity versus diabetes support programme in overweight or obese individuals with T2D over a median of 9.6 years.^124^ Significant improvements in weight loss, glycaemic control and fitness were seen with intensive lifestyle intervention, regardless of sex or ethnicity. However, no difference in the rates of adverse CV outcomes when compared with controls, or between subgroups by sex or ethnicity, which may be due to the overall low event rates and better diabetes management in the entire cohort.

There is no sex-difference in the effect of lifestyle interventions in those with pre-diabetes in terms of reducing risk of diabetes, weight and glucose intolerance.^125^ However, men are less likely to participate in weight management programmes. In a meta-analysis of weight loss intervention studies for obesity (*n* = 13,305), men comprised only 36% of participants although they were 11% more likely to complete the intervention than women.^126^ It also appears that results differ with type of intervention; men tended to lose more weight with a low-calorie diet and defined exercise regime whereas a pharmacological approach was more successful in women.^126^

Lifestyle intervention is proven to be effective in preventing diabetes progression and promoting weight loss in high-risk South Asians with either impaired fasting glucose or impaired glucose tolerance. The Indian Diabetes Prevention Programme and Diabetes Community Lifestyle Improvement Program were RCTs in native Indians with pre-diabetes evaluating culturally tailored lifestyle modification involving exercise and dietary changes, with or without additional metformin, compared with standard care of primarily health advice only.^127,128^ Although there were variations of intervention protocols, both studies reported around a 30% reduction in T2D incidence at 3 years. Other studies have also reported significant weight loss in immigrant South Asians living in the UK.^129^ Longitudinal studies are needed to evaluate the effects of these lifestyle changes on CV outcomes in South Asians.

### Bariatric surgery

Bariatric surgery is an option for those who have failed to lose weight despite lifestyle intervention and pharmacological therapy, although strict eligibility criteria must be met. The three most commonly performed bariatric surgeries are gastric banding, sleeve gastrectomy and roux-en-Y gastric bypass. Bariatric surgery induces significant weight loss and improvement in metabolic risk profile, including remission of T2D. A retrospective study (*n* = 13,722) comparing bariatric surgery with no surgery in T2D reported a 40% reduction in major CV events over median 4-year follow up.^130^ Furthermore, there is improvement in imaging indices of cardiac structure and function, commensurate with the reduction of LV filling pressure.^131^

There appears to be no sex difference in the effectiveness of bariatric surgery. A systematic review comprising 79 women matched to 79 men showed no difference in sustained reduction of BMI and metabolic markers (BP, HbA1c, cholesterol:HDL) at 2 years following bariatric surgery.^132^ Interestingly, a significantly greater proportion of women undergo bariatric surgery than men. Data from the US showed that out of more than 800,000 people undergoing bariatric surgery over a 10-year period, around 80% were women.^133^ Reasons proposed include a greater willingness of women to accept surgery and expectations related to body image. Men undergoing bariatric surgery also had greater co-morbidities, increasing operative risk, which may make surgery less attractive.^133^ Bariatric surgery has been shown to yield positive effects on obese South Asians in observational trials, with the greatest benefit being diabetes remission.^134^

## Conclusion

Diabetes is associated with a relatively greater detrimental impact on CV complications in women than in men. Pathophysiological mechanisms are not fully understood but are likely multifactorial, involving greater risk factor burden in women, effect of sex hormones and different cardiac adaptation to metabolic and haemodynamic stressors. Although South Asians are predisposed to developing T2D earlier compared with white Europeans, associated CVD and mortality among migrant South Asians have declined in recent times and this group are not at higher risk of CV complications than those of white ethnicity. Indeed, South Asians have lower risk of developing PAD and, although data are scarce, do not appear to be at increased risk of heart failure despite more concentric cardiac remodelling. Pharmacological treatment, lifestyle interventions and bariatric surgery appear to have similar effects in both sexes and in ethnic minorities. Further studies in women and ethnic minorities are warranted and, as well as better understanding disease mechanisms, may identify more precise targets for intervention, facilitating precision medicine approaches for the treatment and prevention of complications in T2D.
